# Rate-Aided Clinical Pathology

**Published:** 1914-01-31

**Authors:** 


### RATE-AIDED CLINICAL PATHOLOGY.

CHARGES have been made of late against medical practitioners engaged in contract work under the Insurance Act of inadequacy of treatment, and the most frequently recurring demand of what may presumably be described as "adequate" medical treatment is for those investigations which come under the heading of clinical pathology. For the sake of clearness it may be appropriate to enumerate a few representative examinations which will in the nature of things be called for in any general practice: such, for example, as the various estimations and examinations of the urine; the microscopy of the blood cells; the cytological examinations of the body fluids; bacteriological examinations of throat swabs and sputa; the Widal test for enteric fever; the Wassermann reaction, and the preparation of vaccines. There are, of course, many other investigations which are daily performed in considerable number in any large hospital laboratory, but any of the foregoing may be and should be demanded in general practice without the patient necessarily requiring admission to the wards of an institution.

Now, it is obvious that under the present conditions of medical practice no general practitioner can find the time, even if the ability be granted, for doing such work and yet make a living for himself. Nor is there any reason why he should; such work is essentially of a nature that is better done centrally and collectively. The inadequacy of the existing provision for clinical pathology for the population at large needs not to be laboured. No official provision is made for this work, except to a very limited extent by local authorities, and then only for a few diseases, such as diphtheria.

The only practicable alternatives for bringing about a satisfactory improvement in the conditions of domiciliary treatment appear to be either to extend the pathological facilities at hospitals, or for hospitals and local authorities to combine forces for the necessary additional provision.

In a Memorandum by the medical officer of the Local Government Board on the pathological work undertaken by or on behalf of public health authorities and by voluntary hospitals, the writer points out how variable are the facilities afforded in counties and county boroughs, and even when these exist they have only a very limited application. A portion of the Memorandum is taken up with a detailed return of the work done in the voluntary hospitals, whence it is seen that in the Metropolitan area at least a very large amount of work is yearly carried out, which may legitimately be regarded as part of the burden of the public health authorities, and which, if not carried out by these bodies, might well be subsidised out of the public funds. The Local Government Board has always impressed on medical officers of health the necessity for taking a broad conception of their duties. These are not limited to communicable diseases. In the Orders of the Board the word used is "disease," unqualified by any adjective. Pathological work forms an important means of ascertaining the distribution and character of disease in a district, and thus enabling rational preventive measures to be taken. In order that a satisfactory medical service may be secured throughout the country, facilities for work similar to that now being done in hospitals must be offered to medical practitioners generally. Unfortunately such an extension of pathological work appears to involve the necessity for further legislation. It is even officially regarded as very doubtful whether County Councils are empowered to run bacteriological laboratories at all. But, fortunately, some are already doing so.

